# Dermatomyositis in an Elderly with Respiratory Presentation: A Case Report

**DOI:** 10.31729/jnma.7401

**Published:** 2022-03-31

**Authors:** Srijana Maharjan, Uma Giri, Anushree Jha, Roshan Rijal, Roshani Bista, Andi Tamang Lama

**Affiliations:** 1 Sumeru City Hospital, Pulchowk, Lalitpur, Nepal; 2 Department of Dermatology and Venereology, National Academy of Medical Sciences, Kathmandu, Nepal; 3 Star Hospital, Sanepa, Lalitpur, Nepal; 4 Kathmandu Medical College and Teaching Hospital, Sinamangal, Kathmandu, Nepal

**Keywords:** *case reports*, *dermatomyositis*, *interstitial*, *lung diseases*, *Nepal*

## Abstract

Dermatomyositis is an idiopathic inflammatory disease that affects the skin and proximal skeletal muscles. To our best knowledge, there are very few cases of Dermatomyositis reported in Nepal and almost none with respiratory manifestation. We present to you the case of dermatomyositis complicated with interstitial lung disease of a 74 years old male patient with a three-week history of generalized weakness, weight loss, Gottron's papule, and heliotrope rash, with features of interstitial lung disease such as shortness of breath, fever, and productive cough, later confirmed by highresolution computed tomography. We monitored his serum creatine kinase/creatine phosphokinase level for two consecutive days which showed a progressive increase (281 U/L to 290 U/L). His antinuclear antibody test was positive. He was managed with standard treatment of dermatomyositis. This report might help understand the presentation of disease in our setting which can help in early diagnosis and early initiation of treatment.

## INTRODUCTION

Dermatomyositis is an idiopathic inflammatory disease that affects the skin and proximal skeletal muscles, whose manifestation can be acute (weeks) or chronic (months-years).^1^ The mean age of diagnosis is 40 years, and nearly twice more common in women, with a prevalence rate of one per 100,000 in the general population.^2^ Its diagnosis is aided by lab, clinical, electromyography, and histopathological tests.^1^ It can present with cutaneous/muscular manifestations.^3,4^ Rarely lung involvement manifests as inflammatory interstitial lung disease (ILD) with aspiration pneumonia due to muscular weakness.^5^ We present here a case of dermatomyositis in 74 years male with a respiratory presentation.

## CASE REPORT

**Figure 1:**
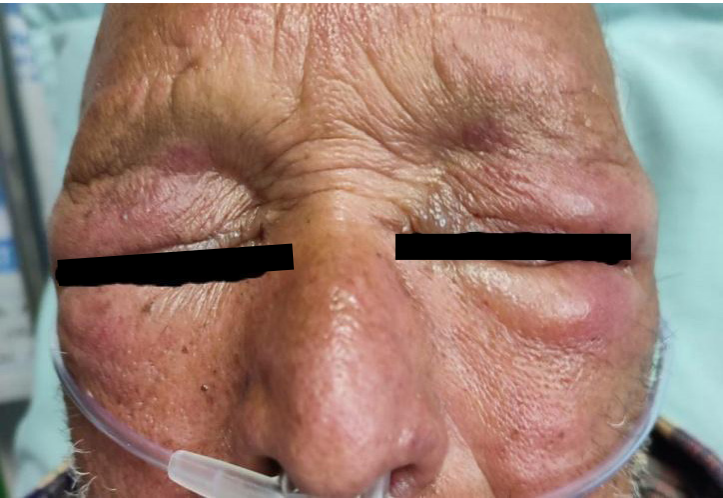
Peri-orbital oedema.

**Figure 2:**
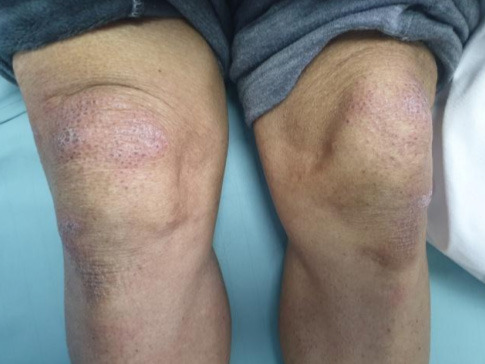
Erythematous macules on the knee.

A 74-year-male patient with a known history of type II diabetes mellitus and hypertension, both under medication, presented to our emergency department with a three-week history of generalized weakness and decreased appetite associated with significant weight loss. He also gave a history of shortness of breath, fever, and productive cough for one week. Examination revealed erythematous rash affecting the upper eyelids with periorbital oedema (Heliotrope Rash) (Figure 1) and erythema over bilateral knees (Figure 2), bilateral elbows and V-sign on the neck.

**Figure 3:**
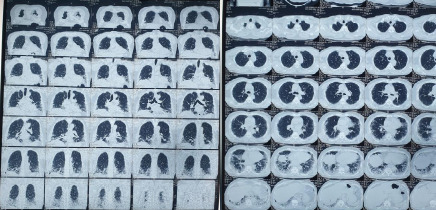
High-resolution computed tomography of the chest.

Chest X-ray revealed bilateral lower zone pneumonia. Reverse-transcriptase polymerase-chain-reaction (RT-PCR) of the oropharyngeal swab for COVID-19 was done which was negative. He was started on antibiotics for pneumonia and on antipyretics for fever. Highresolution computed tomography (HRCT) of the chest was done which suggested interstitial lung disease involving bilateral lower lobes, right middle lobe, and left inferior lingual lobe (Figure 3).

A clinical diagnosis of dermatomyositis complicated by ILD was made. He was admitted and managed with supplemental oxygen, antipyretic, steroid and antibiotics. His saturation level was maintained at the time of admission. His lab parameters at the time of admission showed: Hemoglobin (11.8 g/dl), Total Leukocyte Count (TLC) (9,300/cumm) Differential Leukocyte Count (Neutrophils-81, Lymphocyte 12, Monocyte 6, Eosinophil 1, Basophil 0), and 5 days after admission showed Hb (10.7 g/dl), TLC (12,500/ cumm), Differential Leukocyte Count (Neutrophils-90, Lymphocyte-06, Monocyte-4, Eosinophil-0, Basophil-0).

His Anti-Nuclear Antibody (ANA) test was positive. His electrocardiography (ECHO) showed normal ejection fraction with Grade I Left Ventricle (LV) diastolic dysfunction. Ultrasound of abdomen and pelvis was not remarkable for any significant findings. His thyroid-stimulating hormone (TSH) (2.45 mIU/ml) and renal Function Test was within normal limits and the Liver Function Test showed elevated transaminases level (Serum SGOT and Serum SGPT was 177 U/L and 173 U/L respectively) with normal Serum Alkaline phosphatase level (90 lU/ml). Total Serum Bilirubin- 0.3 mg/dl, Direct Serum Bilirubin- 0.2 mg/dl. His serum Lactate Dehydrogenase (LDH) level had increased by two-fold and is 903 U/L along with elevated C-Reactive Protein (CRP) (quantitative) levels 182 mg/dl. We monitored his serum creatine kinase (CK/CPK) level for two consecutive days which showed a progressive increase, initially it was 281 U/L and increased to 290 U/L the next day. Aldolase was not sent due to the unavailability of the test in Nepal.

After a clinical diagnosis of Dermatomyositis was made, he was started on corticosteroids, Hydrocortisone 100 mg three times a day. Initially patient showed signs of improvement with improvements on his vital signs but on the 9^th^ day of admission, the condition of the patient started to deteriorate and his oxygen saturation started to drop. The patient was shifted to the intensive care unit (ICU), was intubated and kept on the ventilator. On the 2^nd^ day of his ICU admission, the patient was shifted to another hospital where he expired on the same day.

## DISCUSSION

Several types of dermatomyositis reported are classical dermatomyositis, amyopathic dermatomyositis, juvenile dermatomyositis, hypomyopathic dermatomyositis, clinically amyopathic dermatomyositis, and clinical lyamyopathic progressing into classic.^6^ Our patient showed the hallmark cutaneous manifestations of dermatomyositis, as proven by proximal muscle weakness, and laboratory confirmation of myositis.^7^

Muscle weakness and skin findings are usually the main presenting symptoms in dermatomyositis and its onset may be insidious or acute with a waxing and a waning course. Pathognomonic skin findings include Gottron papules (dorsal metacarpophalangeal and interphalangeal joints may show the presence of overlying erythematous or violaceous papules with or without scaling or ulceration and heliotrope rash (violaceous, or an erythematous rash affecting the upper eyelids with or without periorbital edema). Other skin findings are Gottron sign (erythematous macules or patches over the elbows or knees), facial erythema (erythema over the cheeks and nasal bridge involving the nasolabial folds), Shawl sign (erythema over the posterior aspect of the neck, upper back, and shoulders at times, extending to the upper arms), V-sign (ill-defined erythematous macules involving the anterior aspect of the neck and the upper chest).^3^ Our case exhibited the typical findings like erythematous tender papules present in the elbow joint (Gottrons sign) along with classical Heliotrope rash, facial erythema and V sign could also be noted.

In a report the patient showed difficulty in getting up from squatting position, climbing stairs, combing his hair since three months, bilateral weakness, gradual onset, progressive to involve the distal muscles of the upper limb, helitrope rash with rash on cheeks and malar prominence with lab findings of raised ESR, ALT, LDH, CPK and negative ANA.^2^ Similar findings was found in our case but with ANA was positive. They also did Electromyography and muscle biopsy but couldnot be done in our patient due to financial restrictions.

Respiratory manifestation may also be associated with dermatomyositis which includes exertional dyspnea, exercise intolerance, and nonproductive cough due to underlying interstitial lung disease (ILD) with ausculatory presence of bilateral dry crackles and reduced chest movement may be seen due to respiratory muscle weakness.^3^ Similar findings were noted in the patient who came with complains of shortness of breath, cough along with bilateral basal crackles and decreased breath sounds features suggestive of ILD and was later confirmed by HRCT.

In a case reported by Palawisuth S, et al. presented with dermatomyositis with ILD, the patient had fever, rash and muscle pain with weakness.^7^ He also had confluent, symmetric, violaceous patches on his scalp, malar eminences, the “V” area of the upper chest, the upper back, the third and fourth metacarpophalangeal joints, and the extensor tendons of both hands similar to our patient, but he also showed multiple discrete, dull, erythematous papules marked his palms and fingers, which was not seen in our patient.^7^ His noncontrast computed tomography (CT) of the chest revealed predominant ground-glass opacity with traction bronchiectasis in both lower lobes but HRCT in our patient-reported extensive subpleural honeycombing in b/l lower lobes, right middle lobe and left inferior lingular lobe.

A study done by Wu H, et al. reported dermatomyositis patients with ILD had a significantly higher prevalence of heliotrope rash than those without ILD along with a significantly lower prevalence of Gottron's papules than those without ILD and myasthenia, myalgia, difficulty combing the hair climbing stairs, arthralgia, dysphagia and cough were also significantly more common in those with ILD than those without ILD.^5^ Our patient also showed heliotrope rash, myalgia, difficulty climbing stairs and cough.

Autoantibodies test for dermatomyositis diagnosis include Antinuclear antibodies (ANA), myositis specific autoantibodies (MSA), Aminoacyl-transfer (t) ribonucleic acid synthetase (also known as an antisynthetase antibody, Anti-Jo, Anti-Mi2 (directed against-helicase).^3^ This patient has ANA positive but we could not perform Anti Mi2 and Anti Jo antibody tests due to financial restrictions.

Usually patient with dermatomyositis should undergo chest radiography to screen for interstitial lung disease. In any case the patient has respiratory symptoms or abnormal chest X-ray findings, further testing with high-resolution computer tomography (HRCT) of the chest . In addition, pulmonary function tests should be performed. Findings on HRCT suggestive of interstitial lung disease include nodules, fibrosis, linear opacities, honeycombing, or consolidation.^3^ Similar findings was noted on his HRCT which reported extensive subpleural honeycombing in b/l lower lobes, right middle lobe and left inferior lingular lobe.

According to Bohan and Peter criteria, a definite diagnosis requires 4 or more of the following criteria: symmetric proximal weakness, elevated levels of serum muscle enzymes, characteristic electromyography changes, characteristic muscle biopsy changes, and typical skin lesions that may include scalp dermatosis, heliotrope rash, photosensitive poikiloderma, V sign (rash on anterior neck), shawl sign, Gottron papules or Holster sign (rash over lateral hip).^8^ Therefore he could be diagnosed as a case of Dermatomyositis.

Our patient presented as a classical case of dermatomyositis with ILD presentation. Treatment usually consists of immunosuppressants like systemic glucocorticoids, azathioprine or methotrexate. Treatment choices for resistant cases are rituximab, mycophenolate mofetil, calcineurin inhibitors, intravenous immunoglobulin (IVIG), and cyclophosphamide. Cyclophosphamide is usually preferred in cases of rapidly progressive interstitial lung disease.^3^ This patient was given systemic glucocorticoids and his condition initially improved but his ILD worsened and his saturations dropped and the patient expired after about a week of therapy initiation.

ILD is major cause of morbidity and mortality in dermatomyositis. Therefore, timely diagnosis and management is required but still fatality is common.
